# Chemical Diversity of Plant Cyanogenic Glycosides: An Overview of Reported Natural Products

**DOI:** 10.3390/molecules26030719

**Published:** 2021-01-30

**Authors:** Meri Yulvianti, Christian Zidorn

**Affiliations:** 1 Department of Pharmaceutical Biology, Christian-Albrechts-University of Kiel, 24118 Kiel, Germany; myulvianti@pharmazie.uni-kiel.de; 2 Department of Chemical Engineering, Faculty of Engineering, University of Sultan Ageng Tirtayasa, Serang 42124, Indonesia; 3 Indonesia Center of Excellence for Food Security, University of Sultan Ageng Tirtayasa, Serang 42124, Indonesia

**Keywords:** cyanogenic glycosides, plant toxins, specialized natural products

## Abstract

Cyanogenic glycosides are an important and widespread class of plant natural products, which are however structurally less diverse than many other classes of natural products. So far, 112 naturally occurring cyanogenic glycosides have been described in the phytochemical literature. Currently, these unique compounds have been reported from more than 2500 plant species. Natural cyanogenic glycosides show variations regarding both the aglycone and the sugar part of the molecules. The predominant sugar moiety is glucose but many substitution patterns of this glucose moiety exist in nature. Regarding the aglycone moiety, four different basic classes can be distinguished, aliphatic, cyclic, aromatic, and heterocyclic aglycones. Our overview covers all cyanogenic glycosides isolated from plants and includes 33 compounds with a non-cyclic aglycone, 20 cyclopentane derivatives, 55 natural products with an aromatic aglycone, and four dihydropyridone derivatives. In the following sections, we will provide an overview about the chemical diversity known so far and mention the first source from which the respective compounds had been isolated. This review will serve as a first reference for researchers trying to find new cyanogenic glycosides and highlights some gaps in the knowledge about the exact structures of already described compounds.

## 1. Introduction

Like many other plant natural products, cyanogenic glycosides serve as defense agents against herbivores, in this case by releasing toxic hydrogen cyanide after tissue damage. Some plant species are deadly for humans due to their high content of cyanogenic glycosides. For other species, used as staple foods, the content of cyanogenic glycosides requires special modes of preparation in order to detoxify the plants before human consumption. For a third group of plants, the moderate content (or the additional/consumption in moderate amounts) of cyanogenic glycoside makes them highly praised aroma plants (such as almonds in the production of marzipan).

The first cyanogenic glycoside that was isolated from a plant source was amygdalin (**65**) which was obtained from bitter almonds [*Prunus dulcis* (Mill.) D.Webb var. *amara* (DC.) H.Moore] in 1830 [1]. Currently, more than 2500 plant species are known to contain cyanogenic glycosides [2]. To the best of our knowledge, the latest addition to the list of naturally occurring cyanogenic glycosides is prunasin methacrylate (**71**), which was isolated from *Centaurea microcarpa* Coss. & Durieu ex Batt. & Trab. (Asteraceae) in 2018 [3]. In total there are now 112 cyanogenic glycosides reported; 68 cyanogenic glycosides were reported before the year 2000 and 44 cyanogenic glycosides have been reported from 2000 until today.

**Figure 1:**
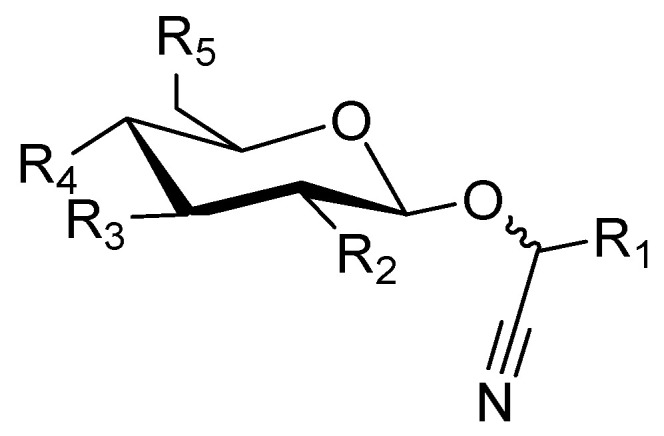
General structure of cyanogenic glycosides.

Cyanogenic glycosides or α-hydroxynitrile glycosides are a unique class of natural products featuring a nitrile moiety, which after enzymatic degradation of the genuine natural product can release hydrogen cyanide (prussic acid). Cyanogenic glycosides consist of two main parts, an aglycone and a sugar moiety. The general structure of cyanogenic glycosides is displayed in Figure 1: R_1_ represents the aglycone part; R_2_, R_3_, R_4_, and R_5_ represent the possible positions of substituents attached to the glucose moiety. The aglycone part consists of a nitrile group linked to an aliphatic, cyclic, aromatic, or heterocyclic moiety. Aglycones of cyanogenic glycosides are biosynthesized starting from one of the following amino acids: phenylalanine, tyrosine, valine, isoleucine, leucine, 2-(2′-cyclopentenyl)-glycine, and 2-(2′-hydroxy-3′cyclopentenyl)-glycine [4]. Additionally, some cyanogenic glycosides (discussed in groups A and B) also feature sulfate groups in their structures. The sugar moiety consists usually of glucose or a substituted glucose moiety. Besides monoglycosides, the most common sugar moieties in cyanogenic glycoside, also di- and triglycosides occur in some compounds. Additional substitutions of all hydroxyl groups of the sugar moiety also exist and thus add to the variety of natural products. In addition to glucose, the following sugars have been reported as parts of cyanogenic glycosides: allose, apiose, arabinose, rhamnose, and xylose.

A sugar moiety is connected to an aglycone via the oxygen linked to the α-carbon atom (relative to the nitrile group). Specific enzymes (β-glucosidases) can easily hydrolyse the resulting structures after tissue damage in the plant. In vitro, hydrolysis is also possible by diluted acids or bases. After splitting-off the sugar moiety from the aglycone by hydrolysis of the β-glycosidic bond, the aglycone can be further degraded, releasing HCN, either spontaneously (in vitro) or facilitated by an additional specific enzyme, (*S*)-hydroxynitrile lyase. This phenomenon is called cyanogenesis, a term first introduced by Henry and Dunstan in 1905 [5].

Some natural cyanogenic glycosides are enantiomers, differing in the stereochemistry of the aglycone, but not the sugar part. Example of such compound pairs are lotaustralin (*R*) (**5**)/epilotaustralin (*S*) (**11**); volkenin (1*R*, 4*R*) (**36**)/epivolkenin (**40**) (1*S*, 4*R*); taraktophyllin (1*R*,4*S*) (**38**)/tetraphyllin B (1*S*,4*S*) (**45**); suberin A (1*R*,2*R*,3*R*,4*R*) (**49**)/suberin B (1*S*,2*S*,3*S*,4*S*) (**51**) and prunasin (*R*) (**54**)/sambunigrin (*S*) (**55**) [6,7].

Cyanogenic glycosides are fascinating natural products, because they have not only a vital role for the plants producing them, but also for other living organisms. The crucial role of cyanogenic glycosides in protecting the plants producing them is particularly crucial at the early stages of plant development. Accordingly, the concentration of cyanogenic glycosides is often higher in seedlings and young leaves than in mature plants [8]. In agriculture, cyanogenic glycosides can also be employed to protect non-source plants from herbivory by spraying preparations containing cyanogenic glycosides (e.g., cassava wastewater obtained in the process of reducing linamarin (**1**) content in cassava, in order to make it safe for human consumption [9]). Moreover, recent results have indicated a potential neuroprotective action of prunasin 2′,3′,4′,6′-tetra-*O*-gallate (**83**) [10].

## 2. Results

The keyword “cyanogenic glycosides” was used to search the literature for references to this particular group of compounds; in this way, we found numerous articles, book chapters, and seminar proceedings, some dating back to the 19th century. These publications were then screened for those, which discussed the isolation and elucidation of cyanogenic glycosides compounds.

In the following sections, all cyanogenic natural products found in the literature are mentioned and displayed in Figure 2, Figure 3, Figure 4, Figure 5, Figure 6, Figure 7, Figure 8, Figure 9, Figure 10, Figure 11, Figure 12, Figure 13, Figure 14, Figure 15, Figure 16, Figure 17 and Figure 18. In Figure 19, abbreviations used in the other figures are explained. In Table 1, the trivial and semi-trivial names (if at all existing) are indicated, along with the compound numbers and the number of the respective figure in which the chemical structure of the compound is presented.

The 112 individual natural products retrieved from the literature, were divided into four groups, based on their aglycones. Group A comprises 33 cyanogenic glycosides with a non-cyclic aliphatic aglycone, group B 20 cyanogenic glycosides featuring cyclopentene or cyclopentane in the aglycone, group C contains 55 cyanogenic glycosides with an aromatic aglycone (some of which are the derivatives of prunasin), and group D consists of four cyanogenic glycosides with a heterocyclic aglycone (pyridinone derivatives). The references cited in this review are, whenever possible, the first articles that reported the isolation of a particular cyanogenic glycoside.

Within each group, compounds have been ordered first by the respective aglycone and then according to the substitution pattern of the respective aglycones and their sugar moieties. Here, compounds with ether bound substituents have been queued before compounds with additional sugar moieties, and these before compounds with acyl-substituents. The (*R*)- and (*S*)-series (if at all applicable or known) of otherwise identical aglycones/compounds have been ordered separately.

### 2.1. Group A: Cyanogenic Glycosides ***1**–**33*** Featuring Acyclic Aliphatic Aglycones

**Figure 2:**
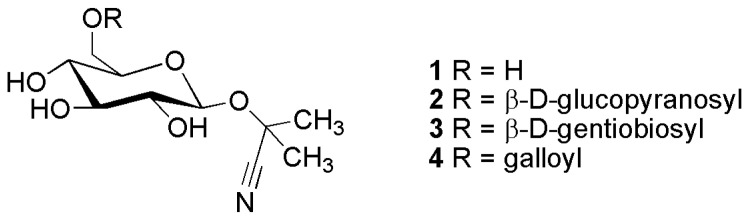
Cyanogenic glycosides group A-1, valine-derived compounds **1**–**4**: linamarin (**1**); linustatin (**2**) and linustatin C (**3**) and linamarin gallate (**4**).

**Figure 3:**
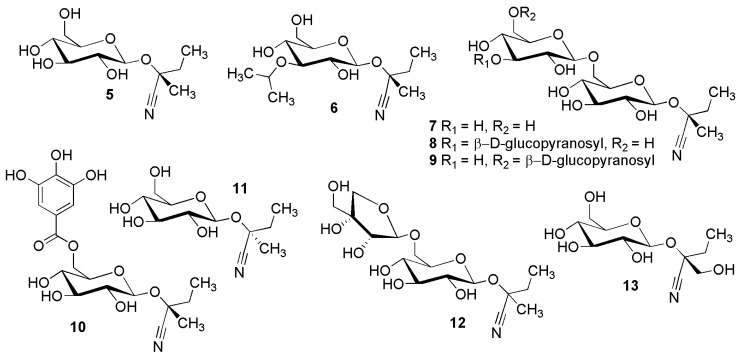
Cyanogenic glycosides group A-2, isoleucine-derived compounds **5**–**13**: (*R*)-lotaustralin (**5**), 2-[(3′-isopropoxy-*O*-β-d-glucopyranosyl)oxy]-2-methylbutanenitrile (**6**), neolinustatin (**7**), linustatin A (**8**), linustatin B (**9**), supinanitriloside C (**10**), (*S*)-epilotaustralin (**11**), 2-[(6-*O*-d-apio-β-d-furanosyl-β-d-glucopyranosyl)oxy]-2-methylbutanenitrile (**12**) and sachaloside V (**13**).

**Figure 4:**
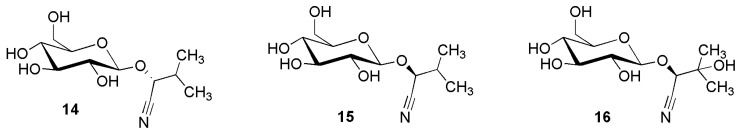
Cyanogenic glycosides group A-3, leucine-derived compounds without a double bond in the aglycone moiety: (*R*)-heterodendrin (**14**), (*S*)-epiheterodendrin (**15**) and (*R*)-3-hydroxyheterodendrin (**16**).

**Figure 5:**
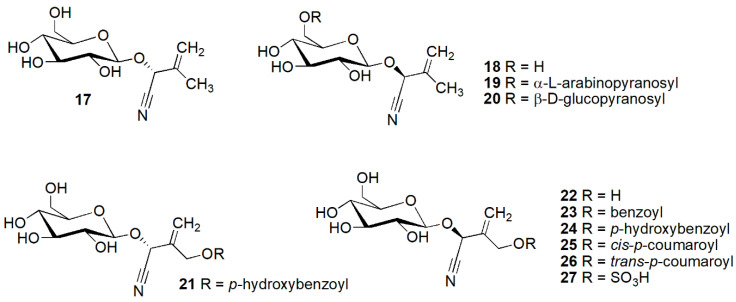
Cyanogenic glycosides group A-4, leucine-derived compounds featuring a methylene group in the aglycone moiety: epiproacacipetalin (**17**), proacacipetalin (**18**), proacaciberin (**19**), proacacipetalin-6′-*O*-β-d-glucopyranoside (**20**), epicardiospermin-5-*p*-hydroxybenzoate (**21**), cardiospermin (**22**), cardiospermin-5-benzoate (**23**), cardiospermin-5-*p*-hydroxybenzoate (**24**), cardiospermin-5-*cis*-*p*-coumarate (**25**) cardiospermin-5-(4-hydroxy)-*trans*-*p*-coumarate (**26**) and cardiospermin-5-sulfate (**27**).

**Figure 6:**
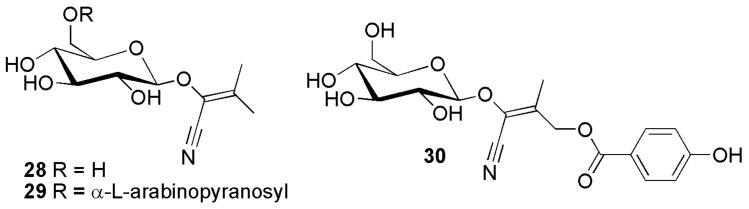
Cyanogenic glycosides group A-5, leucine-derived compounds featuring a double bond in position α relative to the cyano moiety: acacipetalin (**28**), acaciberin (**29**) and isocardiospermin-5-(*p*-hydroxy)benzoate (**30**).

**Figure 7:**
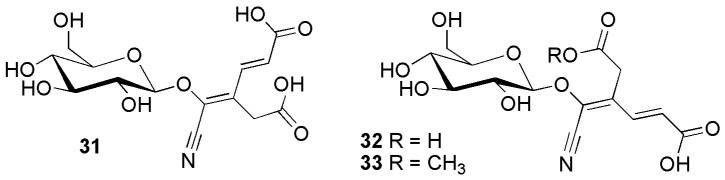
Cyanogenic glycosides group A-6, aliphatic compounds derived from the aromatic amino acid tyrosine **31**–**33**: triglochinin (**31**), isotriglochinin (**32**) and isotriglochinin monomethyl ester (**33**).

This group of cyanogenic glycosides contains compounds derived from the aliphatic amino acids L-valine (Figure 2), L-isoleucine (Figure 3), and L-leucine (Figure 4, Figure 5 and Figure 6) and seco-derivatives from the aromatic amino acid L-tyrosine (Figure 7). Linamarin (**1**) from *Linum usitatissimum* L. (Linaceae) was the first derivative of this group to be isolated (in 1891) [11]. The latest one was isocardiospermin-5-(*p*-hydroxy)benzoate (**30**), which was reported in 2016 [12].

Figure 2 and Figure 3 show the structures of some simple cyanogenic glycosides, which were isolated from *Linum usitatissimum* L.: linamarin (**1**), linustatin (**2**), lotaustralin (**5**), neolinustatin (**7**) [13], linustatin A (**8**), linustatin B (**9**) and linustatin C (**3**) [14] from *Linum usitatissimum* L. (Linaceae). The related compound linamarin gallate (**4**) was reported from *Loranthus micranthus* Hook.f. [as *Loranthus micranthus* (Linn.)] (Loranthaceae) [15]; lotaustralin (**5**) from *Lotus australis* Andrews (Fabaceae) [16]; 2-[(3′-isopropoxy-*O*-β-d-glucopyranosyl)oxy]-2-methylbutanenitrile (**6**) from *Linum grandiflorum* Desf. (Linaceae) [17]; supinanitriloside C (**10**) from *Euphorbia maculata* L. (as *Euphorbia supina* Raf.) (Euphorbiaceae) [18]; epilotaustralin (**11**) from *Triticum monococcum* L. (Poaceae) [19]; 2-[(6-*O*-d-apio-β-d-furanosyl-β-d-glucopyranosyl)oxy]-2-methylbutanenitrile (**12**) from fresh cassava root cortex *Manihot esculenta* Crantz (Euphorbiaceae) [20]; sachaloside V (**13**) from *Rhodiola sachalinensis* Boriss. (Crassulaceae) [21].

The structures of heterodendrin (**14**) and its derivatives are displayed in Figure 4: heterodendrin (**14**) was obtained from the vegetative parts of *Heterodendron oleaefolium* Desf. (Sapindaceae) [22]; epiheterodendrin/dihydroacacipetalin (**15**) [23] and 3-hydroxy-heterodendrin (**16**) [24] from *Acacia sieberiana* DC. var. *woodii* (Burtt Davy) Keay & Brenan (Fabaceae).

Figure 5 features compounds derived from leucine, which contain a methylene group in the aglycone, such as cardiospermin (**22**), derivatives of cardiospermin and some derivatives of acacipetalin. Cardiospermin (**22**) [25] and cardiospermin-5-sulfate (**27**) [26] were isolated from *Cardiospermum hirsutum* Willd. [as *Cardiospermum grandiflorum* Sw. (Sapindaceae)]; cyanogenic glycosides isolated from *Sorbaria sorbifolia* (L.) A.Braun (Rosaceae) such as epicardiospermin-5-*p*-hydroxybenzoate (**21**), (2*S*)-cardiosperminbenzoate (**23**), (2*S*)-cardiospermin-5-*cis*-*p*-coumarate (**25**) [12], (2*S*)-cardiospermin-5-*p*-hydroxy-*trans*-coumarate (**26**) [27]; (2*S*)-cardiospermin-5-*p*-hydroxybenzoate (**24**) from *Sorbaria arborea* C.K. Schneid (Rosaceae) [6,28]. Another derivative of cardiospermin, isocardiospermin-5-*p*-hydroxybenzoate **30** from *Sorbaria sorbifolia* (Rosaceae) [12] is displayed in Figure 6.

Acacipetalin (**28**) (Figure 6) was isolated from *Acacia losiopetala* Oliv. (Fabaceae) [29], proaca-cipetalin (**18**) (Figure 5) from *Acacia sieberiana* and *Acacia hebeclada* DC. (Fabaceae) [30], epiproacacipetalin (**17**) from *Acacia globulifera* Saff. (Fabaceae) [31], proacacipetalin 6′-*O*-β-d-glucopyranoside (**20**) from *Balanophora involucrata* Hook.f. & Thomson (Balanophoraceae) [32] (Figure 5). Acaciberin (**29**) (Figure 6) and proacaciberin (**19**) (Figure 5) were first isolated from *Acacia sieberiana* (Fabaceae) [33].

Figure 7 shows the structures of some unusual ring-cleaved tyrosine derivatives, triglochinin (**31**) and its derivatives. Triglochinin and isotriglochinin (**32**) were first reported from *Triglochin maritimum* L. and *Triglochin palustris* L. [34] and *Alocasia macrorrhizos* (L.) G. Don [35] (Araceae). *Thalictrum aquilegifolium* L. (Ranunculaceae) yielded isotriglochinin monomethyl ester (**33**) [36].

### 2.2. Group B: Cyanogenic Glycosides ***34**–**53*** Derived from Cyclopentenyl Glycine

**Figure 8:**
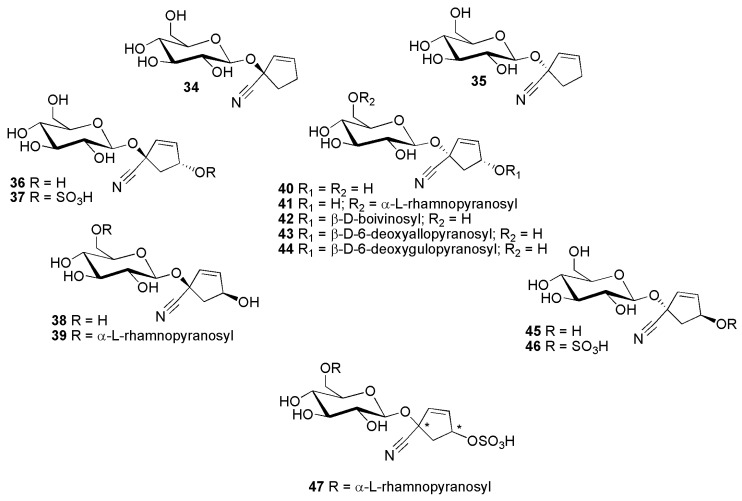
Cyanogenic glycosides group B-1, cyclopentene derivatives with one (compounds **34**–**35**) or two (compounds **36**–**47**) hydroxy groups: (*R*)-deidaclin (**34**), (*S*)-tetraphyllin A (**35**), (1*R*,4*R*)-volkenin (**36**), (1*R*,4*R*)-volkenin sulfate (**37**), (1*R*,4*S*)-taraktophyllin (**38**), (1*R*,4*S*)-6′-*O*-α-l-rhamnopyranosyl taraktophyllin (**39**), (1*S*,4*R*)-epivolkenin/passicoriacin (**40**), 6′-*O*-α-l-rhamnopyranosyl epivolkenin (**41**), passicapsin (**42**) passitrifasciatin (**43**), (1*S*,4*R*)-passibiflorin (**44**), (1*S*,4*S*)-tetraphyllin B (**45**), (1*S*,4*S*)-tetraphyllin B sulfate (**46**) and passicoccin (**47**).

This group currently comprises 20 compounds. The first compound reported from this group was gynocardin (**48**), described in 1905 [37]. The latest one is passiguatemalin (**53**), reported in 2002 [38]. Almost all compounds in this group of cyanogenic glycosides were first isolated from members of the Passifloraceae. Only two were first isolated from the Achariaceae and Flacourtiaceae, respectively. The simplest cyanogenic glycoside structure in this group is deidaclin (**34**) [39], first named deidamin, isolated from *Deidamia clematoides* (C.H.Wright) Harms (Passifloraceae) [40]. Figure 8 shows the structures of deidaclin (**34**), tetraphyllin A (**35**), volkenin (**36**), tetraphyllin B (**45**) and their corresponding sulfate derivatives. Tetraphyllin A (**35**) and tetraphyllin B (**45**) were first isolated from *Tetrapathea tetrandra* (Banks ex DC.) Raoul (Passifloraceae) [41]. Volkenin (**36**) was originally isolated from *Adenia volkensii* Harms (Passifloraceae) as epitetraphyllin B [42], but later renamed volkenin [43]. Volkenin sulfate (**37**) and tetraphyllin B sulfate (**46**) were isolated from *Passiflora caerulea* L. (Passifloraceae) [44]. Taraktophyllin (**38**) and epivolkenin/passicoriacin (**40**) were isolated from *Passiflora coriacea* Juss. (Passifloraceae) [45]. 6′-*O*-α-l-rhamnopyranosyltaraktophyllin (**39**) and 6′-*O*-α-l-rhamnopyranosyl-epivolkenin (**41**) were found in *Hydnocarpus pentandrus* (Buch.-Ham.) Oken (Flacourtiaceae) [46].

Figure 8 also displays the structures of some cyanogenic glycosides group B with unusual sugar residues (6-deoxyallose, initially assigned as rhamnose, in **43**, 6-deoxygulose, initially also assigned as rhamnose, in **44** and **44a**and boivinose in **42**) attached to the glycone part. There had been some confusion about the correct structures of some compounds from this group [47,48,49,50]. Compounds with the trivial names passitrifasciatin (**43**), passibiflorin (**44**) and epipassibiflorin (**44a**) (presumably the C-1 epimer of passibiflorin), were initially assigned other structures regarding the identity (see above) and position [*O*-4′ (compound **43**) or *O*-6′ (compounds **44**, **44a**) of the glucose moiety instead of *O*-4 of the aglycone moiety as in the revised structures] of the unusual sugar moieties [47,48,49,50]. Passitrifasciatin (**43**) was isolated from *Passiflora trifasciata* Lem. (Passifloraceae) [47,48]. Passibiflorin (**44**) and and its presumed 1-epimer epipassibiflorin (**44a**) were isolated from *Passiflora biflora* Lam. and *Passiflora talamancensis* Killip (Passifloraceae) [47,49]. Passicapsin (**42**) was isolated from *Passiflora capsularis* L. (Passifloraceae) [50]. Figure 8 furthermore shows a cyclopentene cyanogenic diglycoside with unresolved stereochemistry in the cyclopentene part of the molecule, containing rhamnose and sulfate moiety, passicoccin (**47**), which was isolated from *Passiflora coccinea* Aubl. (Passifloraceae) [51].

**Figure 9:**
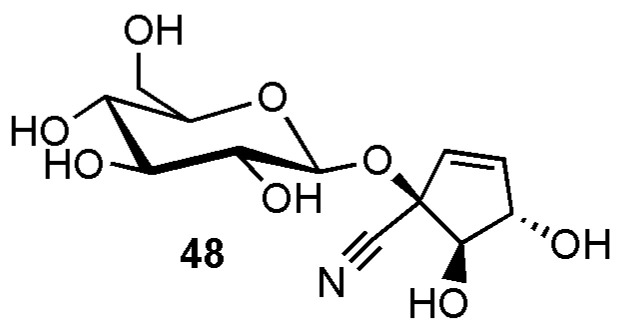
Cyanogenic glycosides group B-2, cyclopentene derivative with three hydroxygroups: (1*R*,4*S*,5*R*)-gynocardin (**48**).

Gynocardin (**48**) (Figure 9), the so far only cyclopentene derivative featuring three hydroxy moieties in the cyclopentene part of the molecule, was isolated from *Gynocardia odorata* R.Br. (Achariaceae) [37].

**Figure 10:**
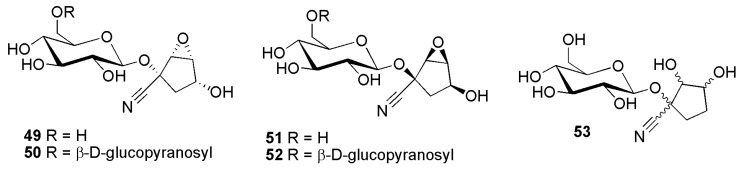
Cyanogenic glycosides group B-3, cyclopentane derivatives **49**–**53**: suberin A (**49**), 6′-*O*-β-d-glucopyranosyl-suberin A (**50**), suberin B (**51**), 6′-*O*-β-d-glucopyranosylsuberin B (**52**) and passiguatemalin (**53**).

Similar to the situation described above, compounds from *Passiflora suberosa* L. depicted in Figure 10 were also isolated in a brief period of time by two competing groups. As again, the first description is not very reliable regarding stereochemistry, we adhere to the publication from Jaroszewski and his team [7] and only mention that Spencer and Seigler [52] were probably the first to isolate compounds **49** and **51** (initially named passisuberosin and epipassisuberosin). However, here we follow Jaroszewski and co-workers [7] and name the compounds (which were first fully elucidated in reference [7]) suberin A (**49**) and B (**51**). The β-d-gentiobiosides additionally described by Spencer and Seigler are tentatively assigned structures **50** and **52**. These should be named 6′-*O*-β-d-glucopyranosylsuberin A (**50**) and 6′-*O*-β-d-glucopyranosylsuberin B (**52**), instead of passisuberosin diglycoside and epipassisuberosin diglycoside, respectively. As a number of compounds allegedly containg rhamnose, isolated by the same authors, proved later to contain other rare sugars, a re-investigation of this compound using modern two-dimensional NMR experiments seems warranted.

Passiguatemalin (**53**) (Figure 10), which could be envisaged as a ring-opened epoxide, was isolated from *Passiflora hahnii* (E.Fourn.) Mast. [as *Passiflora guatemalensis* S.Watson] (Passifloraceae) [38]. Though a full set of spectral data was provided, the stereochemistry of this compound has not been established yet [38].

### 2.3. Group C: Cyanogenic Glycosides ***54**–**108*** Featuring an Aromatic Aglycone

A total of 55 cyanogenic glycosides containing an aromatic aglycone have been described. Amygdalin (**65**) isolated from bitter almond in 1830 was the first cyanogenic glycoside with an aromatic aglycone to be reported [1]. The latest compound is a derivative of prunasin, prunasin methacrylate (**71**), reported in 2018 [3]. An experiment by Fischer in 1895 yielded a derivative compound of amygdalin and was named Fisher’s glycoside [53]. Later on, this glycoside was also isolated from other plant species, such as *Prunus padus* L. (Rosaceae), as prulaurasin (a mixture of prunasin and sambunigrin) [54] and *Prunus serotina* Ehrh. (Rosaceae) [55]. The name prunasin was then introduced in 1912 for compound **54** [56].

**Figure 11:**
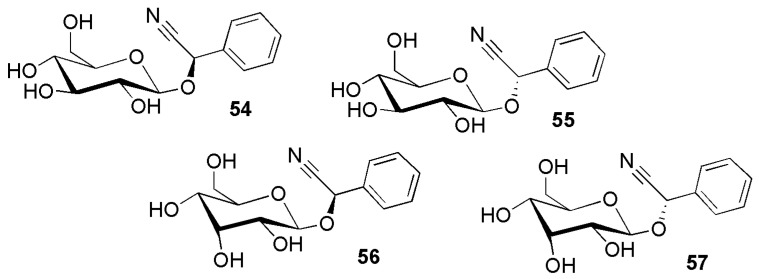
Cyanogenic glycosides group C-1, phenylalanine-derived cyanogenic glycosides **54**–**57** featuring a single, unsubstituted sugar moiety: (*R*)-prunasin (**54**) and (*S*)-sambunigrin (**55**) (2*R*)-passiedulin (**56**) and (2*S*)–β-d-allopyranosyloxy-2-phenylacetonitrile (**57**).

**Figure 12:**
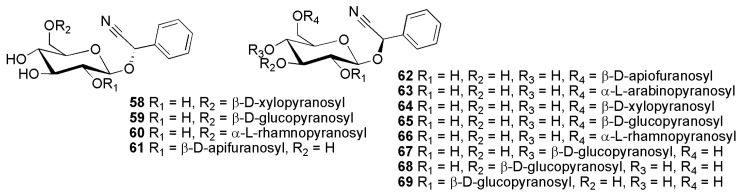
Cyanogenic glycosides group C-2, phenylalanine-derived cyanogenic glycosides featuring an unsubstituted disaccharide in the sugar moiety: (*S*)-epilucumin (**58**), (*S*)-neoamygdalin (**59**), (7*S*)-phenylcyanomethyl 1′-*O*-α-l-rhamnopyranosyl-(1→6)-β-d-glucopyranoside (**60**), (2*S)*-β-d-apio-d-furanosyl-(1→2)-β-d-glucopyranosylmandelonitrile (**61**), oxyanthin (**62**), vicianin (**63**), lucumin (**64**), (*R*)-amygdalin (**65**), (7*R*)-phenylcyanomethyl 1′-*O*-α-l-rhamnopyranosyl-(1→6)-β-d-glucopyranoside (**66**), (*R*)-eucalyptosin B (**67**), (*R*)-eucalyptosin C (**68**), (*R*)-eucalyptosin A (**69**).

**Figure 13:**
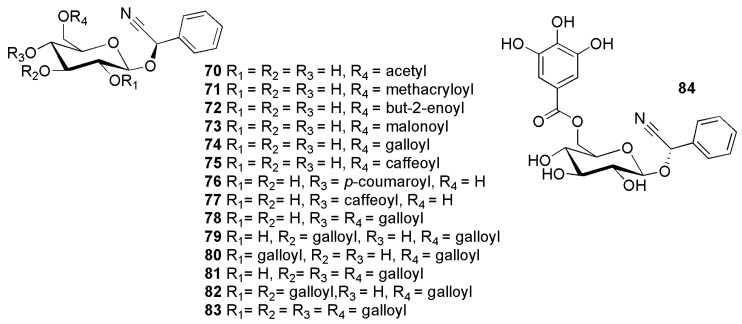
Cyanogenic glycosides group C-3, acyl derivatives of prunasin (**54**) and sambunigrin (**55**): peregrinumcin A (**70**), prunasin 6′-*O*-methacrylate (**71**), prunasin 6′-*O*-trans-2-butenoate (**72**), prunasin-6′-*O*-malonate (**73**) (2*R*)-prunasin 6′-*O*-gallate (**74**), grayanin (**75**), prunasin 4′-*O*-*p*-coumarate (**76**), prunasin 4′-*O*-caffeate (**77**), prunasin 4′,6′-di-*O*-gallate (**78**), prunasin 3′,6′-di-*O*-gallate (**79**), prunasin 2′,6′-di-*O*-gallate (**80**), prunasin 3′,4′,6′-tri-*O*-gallate (**81**), prunasin 2′,3′,6′-tri-*O*-gallate (**82**), prunasin 2′,3′,4′,6′-tetra-*O*-gallate (**83**) and (2*S*)-6′-*O*-galloylsambunigrin (**84**).

**Figure 14:**
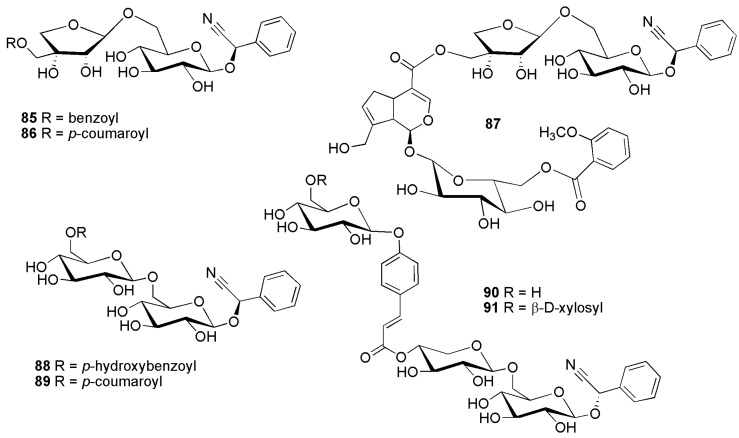
Cyanogenic glycosides group C-4, acyl derivatives of phenylalanine derived cyanogenic glycosides with a disaccharide linked to the aglycone: oxyanthin 5″-*O*-benzoate (**85**), hedyotoside A (**86**), canthium glycoside (**87**), amygdalin-6″-*p*-hydroxybenzoate (**88**), amygdalin-6″-*p*-coumarate (**89**), anthemis glycoside A (**90**) and anthemis glycoside B (**91**).

**Figure 15:**
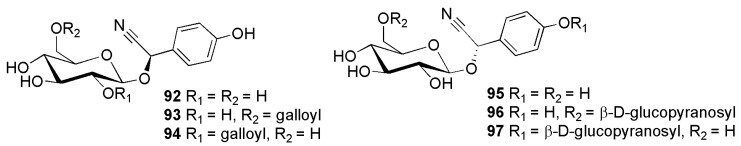
Cyanogenic glycosides group C-5, *para*-hydroxyphenyl derivatives: taxiphyllin (**92**), taxiphyllin 6′-*O*-gallate (**93**), glochidacuminoside D (**94**), dhurrin (**95**), dhurrin 6′-glucoside (**96**) and proteacin (**97**).

**Figure 16:**
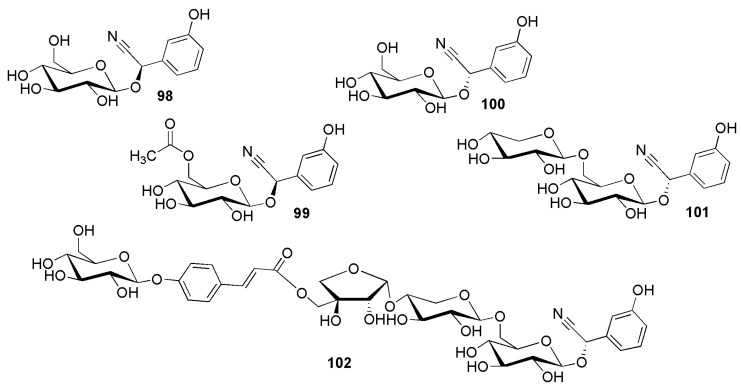
Cyanogenic glycosides group C-6, *meta*-hydroxyphenyl derivatives: (2*R*)-holocalin (**98**), (2*R*)-holocalin acetate (**99**), (2*S*)-zierin (**100**), (2*S*)-zierinxyloside (**101**) and xeranthin (**102**).

**Figure 17:**
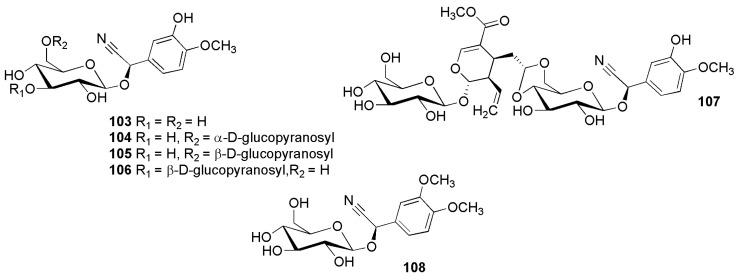
Cyanogenic glycosides group C-7, 3-hydroxy-4-methoxyphenyl and 3,4-dimethoxyphenyl derivatives: hydracyanoside A (**103**) (2*R*)-2-[α-d-glucopyranosyl-(1→6)-β-d-glucopyranosyloxy]-2-(3-hydroxy-4-methoxyphenyl) acetonitrile (**104**), hydracyanoside B (**105**), hydracyanoside C (**106**), hydracyanoside D (**107**) and (2*R*)-2-(β-d-gluco-pyranosyloxy)-2-(3,4-dimethoxyphenyl) acetonitrile (**108**).

To organize the high diversity of aromatic cyanogenic glycosides, these were, somewhat superficially, subdivided in the following groups: compounds derived from phenylalanine, which feature only a single, unsubstituted sugar moiety (Figure 11), phenylalanine-derived cyanogenic glycosides with a disaccharide linked to the aglycone (Figure 12), acyl derivatives of prunasin and sambunigrin (Figure 13), complex acyl derivatives of phenylalanine with a disaccharide linked to the aglycone (Figure 14), *para*-hydroxyphenyl cyanogenic glycosides (Figure 15), *meta*-hydroxyphenyl cyanogenic glycosides (Figure 16), 3-hydroxy-4-methoxyphenyl and 3,4-dimethoxyphenyl cyanogenic glycosides (Figure 17).

Prunasin (**54**) as the most widely distributed cyanogenic glycoside, has been isolated from 14 different plant families: Adoxaceae, Asteraceae, Caricaceae, Dennstaedtiaceae, Fabaceae, Lamiaceae, Melastomataceae, Myrtaceae, Passifloraceae, Penaeaceae (Olinieae), Polypodiaceae, Rosaceae, Rubiaceae, and Salicaceae (Figure 11). In addition, there are more derivatives of prunasin than for any other basic cyanogenic glycosides structures. The (*2S*)-epimer of prunasin (**54**), sambunigrin (**55**), was first isolated from the leaves of *Sambucus nigra* L. (Adoxaceae) [57]. The allopyranosides passiedulin (**56**) [58] and (2*S*)–β-d-allopyranosyloxy-2-phenylacetonitrile (**57**) [59] were both reported from *Passiflora edulis* Sims (Passifloraceae).

Cyanogenic diglycosides with two different sugar moieties encompass (7*R*)-phenylcyanomethyl 1′-*O*-α-l-rhamnopyranosyl-(1→6)-β-d-glucopyranoside (**66**) from *Passiflora edulis* Sims (Passifloraceae) [60]. This compound was later also isolated from dried vines of *Passiflora quadrangularis* L. (Passifloraceae) along with its epimer (7*S*)-phenylcyanomethyl 1′-*O*-α-l-rhamnopyranosyl-(1→6)-β-d-glucopyranoside (**60**) [61]. Vicianin (**63**) was first reported from *Vicia sativa* L. subsp. *nigra* (L.) Ehrh. (as *Vicia angustifolia* L.) (Fabaceae) [62]. Lucumin (**64**) was isolated from *Manilkara zapota* (L.) P.Royen [as *Lucuma mammosa* (L.) C.F.Gaertn.] [63], while epilucumin (**58**), anthemis glycoside A (**90**) and anthemis glycoside B (**91**) were found in *Anthemis retusa* Delile (as *Anthemis cairica* Vis.) and *Cota altissima* (L.) J.Gay (as *Anthemis altissima* L.) (Asteraceae) [64].

Cyanogenic glycosides featuring an apiose moiety are e.g., oxyanthin (**62**) and oxyanthin 5″-*O*-benzoate (**85**) from *Psydrax livida* (Hiern) Bridson and *Oxyanthus pyriformis* (Hochst.) Skeels subsp. *pyriformis* (Rubiaceae) [65]; 2*S*-β-d-apio-d-furanosyl-(1→2)-β-d-glucopyranosylmandelonitrile (**61**) from *Sambucus nigra* L. (Adoxaceae) [66]; hedyotoside A (**86**) from *Hedyotis scandens* Roxb. (Rubiaceae) [67]; canthium glycoside (**87**) from *Psydrax schimperiana* (A.Rich.) Bridson (as *Canthium schimperianum* A.Rich.) (Rubiaceae) [68];

The cyanogenic diglucoside amygdalin (**65**) was first reported from bitter almonds, *Prunus dulcis* (Mill.) D.A.Webb [as *Prunus amygdalus* (DC.) Focke var. *amara*] (Rosaceae) in 1830 [1]. The epimeric compound neoamygdalin (**59**) has been reported from the racemic mixture since 1903 [69]. Eucalyptosin A (**69**) was first isolated from *Eremophila maculata* P.J.Müll. (Scrophulariaceae) [70]. Eucalyptosin A (**69**) [(*R*)-mandelonitrile β-sophoroside], together with eucalyptosin B (**67**) (mandelonitrile β-cellobioside), and eucalyptosin C (**68**) (mandelonitrile β-laminaribioside) have been isolated from *Eucalyptus camphora* F.Muell. ex R.T.Baker (Myrtaceae) [71]. Two amygdalin derivatives, amygdalin-6″-(4-hydroxy)benzoate (**88**) and amygdalin-6″-*p*-coumarate (**89**) were first reported from *Merremia dissecta* (Jacq.) Hall.f. (Convolvulaceae) [72].

Prunasin derivatives peregrinumcin A (**70**) from *Dracocephalum peregrinum* L. (Lamiaceae) [73]; prunasin 6′-*O*-methacrylate (**71**) from *Centaurea microcarpa* Coss. & Durieu ex Batt. & Trab. (Asteraceae) [3]; prunasin 6′-*O*-*trans*-2-butenoate (**72**) from *Centaurea aspera* L. var. subinermis DC. (Asteraceae) [74]; prunasin-6′-*O*-malonate (**73**) from *Merremia dissecta* (Jacq.) Hall.f. (Convolvulaceae) [75], *Lotononis fruticoides* B.-E.van Wyk and *Lotononis falcata* (E.Mey.) Benth. (Fabaceae) [76] are displayed in Figure 13 with related compounds.

Gallate derivatives of prunasin encompass prunasin 6′-*O*-gallate (**74**), prunasin 4′,6′-di-*O*-gallate (**78**), prunasin 3′,6′-di-*O*-gallate (**79**), prunasin 2′,6′-di-*O*-gallate (**80**), prunasin 3′,4′,6′-tri-*O*-gallate (**81**), prunasin 2′,3′,6′-tri-*O*-gallate (**82**), prunasin 2′,3′,4′,6′-tetra-*O*-gallate (**83**) from *Phyllagathis rotundifolia* (Jack) Blume (Melastomaceae) [77], and 6′-*O*-galloylsambunigrin (**84**) from *Elaeocarpus sericopetalus* F.Muell. (Elaeocarpaceae) [78].

Grayanin (**75**) was found in *Prunus grayana* Maxim. (Rosaceae) [79]. Coumaryl and caffeoyl derivatives of prunasin are prunasin 4′-*O*-*p*-coumarate (**76**) and prunasin 4′-*O*-caffeate (**77**) and were reported from *Microlepia strigosa* (Thunb.) C.Presl (Dennstaedtiaceae) [80].

Taxiphyllin (**92**) was first isolated as phyllanthin from *Phyllanthus gunnii* Hook.f. (as *Phyllanthus gastroemii* Müll.Arg.) (Phyllantaceae) [81]. The compound was later re-named to taxiphyllin, because the same compound had been isolated from *Taxus canadensis* Marshall (Taxaceae) [82]. Dhurrin (**95**) was first isolated from *Sorghum bicolor* (L.) Moench (as *Sorghum vulgare* Pers.) (Poaceae); the name dhurrin originates from the Egyptian name of the species, “dhurra shirshabi” [83]. Dhurrin 6′-glucoside (**96**) was isolated from *Sorghum bicolor* (Graminae) [84] and *Polyscias australiana* (F.Muell.) Philipson (Araliaceae) [85]. Taxiphyllin 6′-*O*-gallate (**93**) was isolated from *Syzygium samarangense* (Blume) Merr. & L.M.Perry (Myrtaceae) [86]. Glochidacuminoside D (**94**) was isolated from the leaves of *Glochidion acuminatum* Müll.Arg. (Euphorbiaceae) [87]. Proteacin (**97**) was reported from shoots of *Thalictrum aquilegifolium* L. (Ranunculaceae) [88].

The structures of *meta*-hydroxyphenyl cyanogenic glycosides (Figure 16) furthermore include holocalin (**98**) from *Holocalyx balansae* Micheli (Fabaceae) [89], holocalin acetate (**99**) from *Sambucus nigra* L. (Adoxaceae) [66]; zierin (**100**) from *Zieria lævigata* Sm. (Rutaceae) [90], and zierinxyloside (**101**) from *Xeranthemum cylindraceum* Sm. (Asteraceae-Cardueae) [91]. One of the most complex cyanogenic glycosides, xeranthin (**102**) was reported from *Xeranthemum cylindraceum* Sm. (Asteraceae-Cardueae) [92].

Figure 17 shows the structures of cyanogenic glycosides isolated from *Hydrangea macrophylla* (Thunb.) Ser. (Saxifragaceae), hydracyanoside A (**103**), (*2R*)-2-[α-d-glucopyranosyl(1→6)-β-d-glucopyranosyloxy]-2-(3-hydroxy-4-methoxyphenyl) aceto- nitrile (**104**) and (*2R*)-2-(β-d-glucopyranosyloxy)-2-(3,4-dimethoxyphenyl)] acetonitrile (**108**) [93], hydracyanoside B (**105**), hydracyanoside C (**106**) [94], hydracyanoside D (**107**) [95].

### 2.4. Group D: Cyanopyridone Glycosides ***112**–**115***

**Figure 18:**
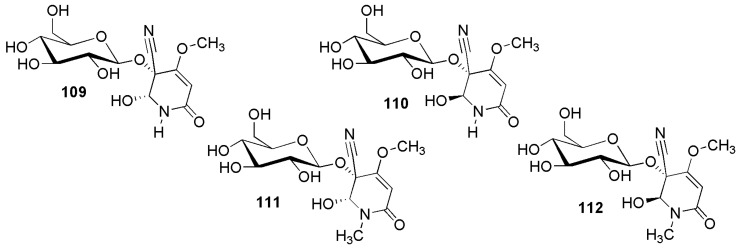
Cyanogenic glycosides group D, dihydropyridone derivatives: (5*R*,6*R*)-epinoracalyphin (**109**), (5*R*,6*S*)-nor-acalyphin (**110**) (5*R*,6*R*)-epiacalyphin (**111**) and (5*R*,6*S*)-acalyphin (**112**).

Cyanopyridone glycosides (Figure 18) encompass only four compounds: acalyphin (**112**), epiacalyphin (**111**), noracalyphin (**110**) and epinoracalyphin (**109**). Acalyphin was first isolated in 1937 from *Acalypha indica* L. (Euphorbiaceae), but its structure could not be determined at the time [96]. Complete structure elucidation and naming of acalyphin was accomplished in 1982 with material isolated from the same species [97]. Acalyphin (**112**) also has been isolated from *Acalypha fruticosa* Forssk. [98]. A more detailed study in 2009 of *Acalypha indica* yielded also epiacalyphin (**111**), noracalyphin (**110**) and epinoracalyphin (**109**) [99].

**Figure 19:**
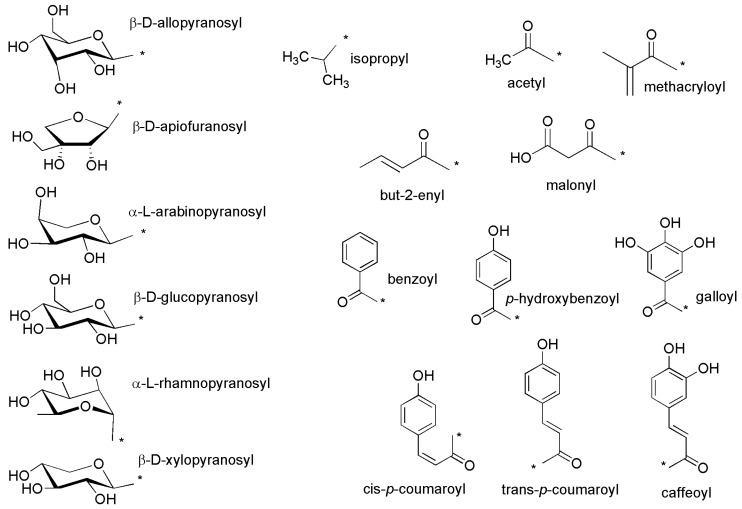
Substituents usually not fully drawn in Figure 2, Figure 3, Figure 4, Figure 5, Figure 6, Figure 7, Figure 8, Figure 9, Figure 10, Figure 11, Figure 12, Figure 13, Figure 14, Figure 15, Figure 16, Figure 17 and Figure 18.

## 3. Bioactivity

Cyanogenic glycosides are foremost toxins, protecting the plant producing them from herbivores [2]. Recent studies on the role of cyanogenic glycosides in plant development have in addition revealed a function of cyanogenic glycosides as a nitrogen source for developmental processes and in playing a role in the adaption to environmental challenges [4].

Cyanide released from edible plants containing cyanogenic glycosides have been reported to cause adverse health effects in humans, e.g., irreversible paralytic disorder, tropical ataxic neuropathy, optical atrophy, angular stomatitis, sensory gait ataxia, neurosensory deafness, goitre, and cretinism [100].

Due to their presence in numerous edible plants, there are many reports dealing with the bioactivities of linamarin (**1**) and amygdalin (**65**). For decades, amygdalin (**65**) has been investigated for its potential application in cancer treatment [101,102]. Targeted cancer therapy, such as suicide gene therapy, antibody-directed enzyme prodrug therapy (ADEPT), and nanoporous imprinted polymers (nanoMIPs) gave particularly promising results [103]. While amygdalin (**65**) has mainly been investigated as a potential anticancer agent, studies focusing on linamarin (**1**) are mainly related to agriculture. Here, linamarin (**1**), a side product from making cassava safe for human consumption, has been tested as herbicides and bio-pesticides [104].

Additionally, some potential applications of cyanogenic glycosides in medicine have recently been patented, e.g., the use of epicardiospermin-5-*p*-hydroxybenzoate (**21**) [105], (2*S*)-cardiospermin-5-benzoate (**23**) [106], and (2*S*)-cardiospermin-5-*cis*-*p*-coumarate (**25**) [107] against rheumatoid arthritis.

## 4. Materials and Methods

Literature was searched for cyanogenic glycosides using Google Scholar, PubChem, Reaxys, and SciFinder. Keywords were “cyanogenic glycosides”, “cyanogenesis” as well as individual names of known cyanogenic glycosides. Names of the known cyanogenic glycosides compounds often led to articles reporting the isolation of related structures. All of the references in this review were then accessed from the homepages of their respective journals and for older articles, published between the years 1800–1930, from the Biodiversity Heritage Library website (https://www.biodiversitylibrary.org). After collecting the articles, structures of individual cyanogenic glycosides were then sorted as described in the results section, not only based on the precursor in the respective biosynthetic pathways [108], but also based on superficial chemical similarity (e.g., group A6).

## 5. Conclusions

The overview provided above shows that currently, 112 distinct cyanogenic glycosides are known from the plant kingdom. This is considerably more than the current literature estimate of about 60 different compounds [109]. Cyanogenic glycosides with an aromatic aglycone are the most diverse group with 55 individual natural products. The most complex structure containing a cyanogenic glycoside moiety is canthium glycoside (**87**) from *Psydrax schimperiana* (A.Rich.) Bridson (as *Canthium schimperianum* A.Rich.) (Rubiaceae). This compound, which also features an iridoid moiety, has a molecular formula of C_43_H_51_NO_21_. In contrast linamarin (**1**) as the simplest known cyanogenic glycoside has a molecular formula of only C_10_H_17_NO_6_. Many of the natural products compiled above, have so far only been reported form a single source, while others are unusually widely distributed in the plant kingdom. Some of the rare compounds have been isolated in times, when structure elucidation of complex natural products was more difficult than today and some re-assignments regarding exact positions of sugar moieties and stereochemistry seem inevitable, when these compounds will be re-investigated. Looking at the enormous possibilities how cyanogenic glycosides, such as e.g., prunasin (**54**) can be incorporated into more complex natural products, makes it intuitively clear that many more natural products encompassing a cyanogenic glycoside moiety still can be discovered.

This review is intended as a guide to get a quick overview, which compounds have already been described and where in the plant kingdom to look for potentially new natural products. The lack of detailed bioactivity reports on any other cyanogenic glycosides other than amygdalin and linamarin are also potential research topics.

## Figures and Tables

**Table 1:** Cyanogenic glycosides covered in this review.

Trivial/Semi-Trivial Name	Figure	Nr.	Trivial/Semi-Trivial Name	Figure	Nr.
**Aliphatic Compounds**			**Aromatic Compounds**		
Linamarin	2	**1**	prunasin	11	**54**
Linustatin	2	**2**	sambunigrin	11	**55**
Linustatin C	2	**3**	passiedulin	11	**56**
Linamarin gallate	2	**4**	-	11	**57**
(*R*)-lotaustralin	3	**5**	epilucumin	12	**58**
-	3	**6**	neoamygdalin	12	**59**
neolinustatin	3	**7**	-	12	**60**
linustatin A	3	**8**	-	12	**61**
linustatin B	3	**9**	oxyanthin	12	**62**
supinanitriloside C	3	**10**	vicianin	12	**63**
(*S*)-epilotaustralin	3	**11**	lucumin	12	**64**
-	3	**12**	amygdalin	12	**65**
sachaloside V	3	**13**	-	12	**66**
heterodendrin	4	**14**	eucalyptosin B	12	**67**
epiheterodendrin	4	**15**	eucalyptosin C	12	**68**
3-hydroxyheterodendrin	4	**16**	eucalyptosin A	12	**69**
epiproacacipetalin	5	**17**	peregrinumcin A	13	**70**
proacacipetalin	5	**18**	prunasin 6’-*O*-methacrylate	13	**71**
proacaciberin	5	**19**	prunasin 6’-*O*-trans-2-butenoate	13	**72**
proacacipetalin-6’-*O*-β-d-glucoside	5	**20**	prunasin-6’-*O*-malonate	13	**73**
epicardiospermin-5-*p*-hydroxybenzoate	5	**21**	prunasin 6’-*O*-gallate	13	**74**
cardiospermin	5	**22**	grayanin	13	**75**
cardiospermin-5-benzoate	5	**23**	prunasin 4’-*O*-*p*-coumarate	13	**76**
cardiospermin-5-*p*-hydroxybenzoate	5	**24**	prunasin 4’-*O*-caffeate	13	**77**
cardiospermin-5-*cis*-coumarate	5	**25**	prunasin 4’,6’-di-*O*-gallate	13	**78**
cardiospermin-5-*trans*-*p*-coumarate	5	**26**	prunasin 3’,6’-di-*O*-gallate	13	**79**
cardiospermin-5-sulfate	5	**27**	prunasin 2’,6’-di-*O*-gallate	13	**80**
acacipetalin	6	**28**	prunasin 3’,4’,6’-tri-*O*-gallate	13	**81**
acaciberin	6	**29**	prunasin 2’,3’,6’-tri-*O*-gallate	13	**82**
isocardiospermin-5-*p*-hydroxybenzoate	6	**30**	prunasin 2’,3’,4’,6’-tetra-*O*-gallate	13	**83**
triglochinin	7	**31**	6’-*O*-galloylsambunigrin	13	**84**
isotriglochinin	7	**32**	oxyanthin 5’’-*O*-benzoate	14	**85**
isotriglochininmonomethylester	7	**33**	hedyotoside A	14	**86**
deidaclin	8	**34**	canthium glycoside	14	**87**
tetraphyllin A	8	**35**	amygdalin- 6’’-*p*-hydroxybenzoate	14	**88**
volkenin	8	**36**	amygdalin-6’’-*p*-coumarate	14	**89**
volkenin sulfate	8	**37**	anthemis glycoside A	14	**90**
taraktophyllin	8	**38**	anthemis glycoside B	14	**91**
6’-*O*-α-l-rhamnosyltaraktophyllin	8	**39**	taxiphyllin	15	**92**
epivolkenin/passicoriacin	8	**40**	taxiphyllin 6’-*O*-gallate	15	**93**
6’-*O*-α-l-rhamnopyranosylepivolkenin	8	**41**	glochidacuminoside D	15	**94**
passicapsin	8	**42**	dhurrin	15	**95**
passitrifasciatin	8	**43**	dhurrin 6’-glucoside	15	**96**
passibiflorin	8	**44**	proteacin	15	**97**
tetraphyllin B	8	**45**	holocalin	16	**98**
tetraphyllin B sulfate	8	**46**	holocalin acetate	16	**99**
passicoccin	8	**47**	zierin	16	**100**
gynocardin	9	**48**	zierinxyloside	16	**101**
suberin A	10	**49**	xeranthin	16	**102**
6′-*O*-β-d-glucopyranosylsuberin A	10	**50**	hydracyanoside A	17	**103**
suberin B	10	**51**	-	17	**104**
6′-*O*-β-d-glucopyranosylsuberin B	10	**52**	hydracyanoside B	17	**105**
passiguatemalin	10	**53**	hydracyanoside C	17	**106**
			hydracyanoside D	17	**107**
			-	17	**108**
			**Dihydropyridone derivatives**		
			epinoracalyphin	18	**109**
			noracalyphin	18	**110**
			epiacalyphin	18	**111**
			acalyphin	18	**112**
